# Artificial physics engine for real-time inverse dynamics of arm and hand movement

**DOI:** 10.1371/journal.pone.0295750

**Published:** 2023-12-13

**Authors:** Mykhailo Manukian, Serhii Bahdasariants, Sergiy Yakovenko

**Affiliations:** 1 Faculty of Applied Science, Ukrainian Catholic University, Lviv, Ukraine; 2 Department of Human Performance—Pathophysiology, Rehabilitation, and Performance, School of Medicine, West Virginia University, Morgantown, West Virginia, United States of America; 3 Department of Neuroscience, School of Medicine, West Virginia University, Morgantown, West Virginia, United States of America; 4 Rockefeller Neuroscience Institute, School of Medicine, West Virginia University, Morgantown, West Virginia, United States of America; 5 Mechanical and Aerospace Engineering, Benjamin M. Statler College of Engineering and Mineral Resources, West Virginia University, Morgantown, West Virginia, United States of America; 6 Department of Biomedical Engineering, Benjamin M. Statler College of Engineering and Mineral Resources, West Virginia University, Morgantown, West Virginia, United States of America; Georgia State University, UNITED STATES

## Abstract

Simulating human body dynamics requires detailed and accurate mathematical models. When solved inversely, these models provide a comprehensive description of force generation that considers subject morphology and can be applied to control real-time assistive technology, for example, orthosis or muscle/nerve stimulation. Yet, model complexity hinders the speed of its computations and may require approximations as a mitigation strategy. Here, we use machine learning algorithms to provide a method for accurate physics simulations and subject-specific parameterization. Several types of artificial neural networks (ANNs) with varied architecture were tasked to generate the inverse dynamic transformation of realistic arm and hand movement (23 degrees of freedom). Using a physical model, we generated representative limb movements with bell-shaped end-point velocity trajectories within the physiological workspace. This dataset was used to develop ANN transformations with low torque errors (less than 0.1 Nm). Multiple ANN implementations using kinematic sequences solved accurately and robustly the high-dimensional kinematic Jacobian and inverse dynamics of arm and hand. These results provide further support for the use of ANN architectures that use temporal trajectories of time-delayed values to make accurate predictions of limb dynamics.

## Introduction

The accurate and fast models of body dynamics help to unravel neuromechanical interactions within movement control pathways [1]. The latest demand is for the implementation of body dynamics within intuitive human-machine interfaces to increase their generality and stability. One of the main challenges for this approach is the speed–accuracy tradeoff. Complex segmental dynamics is computationally intense for realistic body biomechanics. Another problem is the subject-specific dynamics that requires morphometric measurements and skilled model adaptation. In the context of the hand and arm, a realistic musculoskeletal model consists of about 23 degrees of freedom (DOF) actuated by 26 force-generating musculotendon units scaled by limb segment geometry. This complexity is a challenge for real-time simulations and can benefit from approximations of musculoskeletal transformations [2, 3]. What remains is the computation of limb dynamics that is generally performed with the classical physics [4, 5].

Artificial neural networks (ANNs) were previously applied to solve this problem by approximating the input-output transformations from neural signals to the intended movement [6–9]. However, these ANN-based solutions have not exceeded the robust decoding of several (up to 10) DOFs even within limited, stereotypical tasks and postures [9]. The potential reason for the limited success is the expectation that the neural-to-motion transformation can be solved *without* the explicit description of segment dynamics and interactions with external objects. For example, in human brain-computer interface (BCI) that transforms neural activity into intended movement, algorithms trained only on the kinematics of reaching actions are challenged by the presentation of physical objects [10]. The neural activity likely expresses nonlinearities of musculoskeletal actions [11] and their dependency on dynamical demands of object manipulation, which disrupts simplistic statistical decoders. Theoretically, increasing decoder complexity and training with large datasets can mitigate this problem. However, the exponential increase in both size of the training dataset and the required training duration may be prohibitive for realistic transformations. While ANN formulation is attractive for developing neural interfaces for movement and motor learning, it has fallen short of capturing motor complexity.

In this study, we continue the effort of developing a theoretical framework that employs ANNs guided by signal processing in BCI to define a full transformation from neural inputs to decoded physical actions. The main contribution of this work is the methodology for separately solving spatiotemporal dynamics corresponding to limb physics that can be combined with our previous solutions of musculoskeletal relationships [2, 3]. The ANN-based solutions of spatiotemporal characteristics in dynamical systems have been recently shown for the relatively simple Lorenz system [12]. The representation of accurate arm dynamics with the ANN formulation is the focus of this study. Preliminary results of this work were published in a thesis form [13].

## Materials & methods

### Kinematic and kinetic datasets

**Fig 1:**
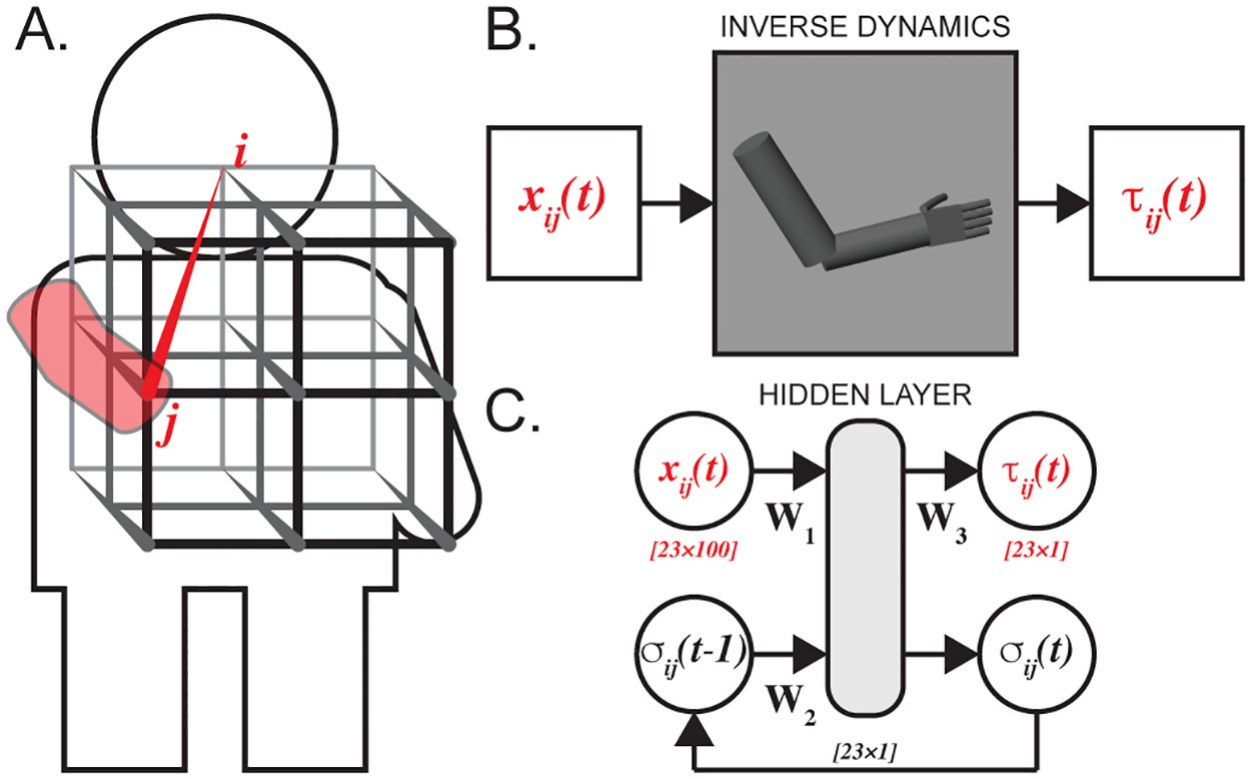
Dataset and modeling approach. The dataset comprised kinematics and kinetics of arm reaching movements within a 3D grid, based on postures recorded from a single representative able-bodied adult. A graphic depicts the relative location of the grid, and a 3D-rendered image illustrates one of the representative postures of the participant. (B) An inverse dynamic model of a segmented arm and hand was applied to simulate the kinetics and kinematics of the movements between the vertices of the grid. (C) Recurrent neural networks (RNNs) were employed to approximate the transformation from kinematics to kinetics: *x*_*ij*_ is input kinematic and *τ*_*ij*_ is output kinetic signals; *σ*_*ij*_ are outputs of the hidden layer recirculated to compute kinetics on the next time step; ***W***_**1–3**_ are weight matrices.

Joint kinetics and kinematics represent reaching movements between a selection of start and finish positions within the arm workspace. The movements were simulated using an assumption of a linear trajectory with smooth, bell-shaped speed profiles [14] passed through a realistic dynamic model to compute joint torques to create training and testing datasets for ANN transformations. Similar to [15], the physiological workspace was covered with a 3x3x3 grid of vertices to generate a representative set of upper-limb movements between them. All combinatorial combinations of movements (Fig 1) were simulated so that each movement represented end-point trajectory as a dynamical set of angular kinematics and corresponding joint torques [16] for three movement durations (0.5, 1.0, 2.0 s). The initial and final joint postures for each vertex in the grid were based on a set of observations from a representative morphometry in a adult (male, 25 y.o., 1.8 m, 72.6 kg). The grid position was aligned relative to the reaching or ipsilateral side. The first column of three positions corresponded to the outstretched arm with elbow extended and shoulder flexion-extension angle at -45º, 0º (*j*-vertex in Fig 1A), and 45º relative to the horizonal. The column of most distal points was selected at the corresponding three points but with shoulder abduction placing finger tips in front of the contralateral shoulder. The proximal vertices were placed at the same vertical levels as the distal vertices but only 10 cm anterior from the body. The middle positions were chosen midway within the medio-lateral and anterior-posterior directions. The set of joint kinetics was computed at 1 ms timestep using a custom 23 DOF biomechanical model of the human arm and hand (Fig 1B), formulated in Simscape Multibody (Simulink R2022a, MathWorks, Inc.) and scaled to a representative subject morphometry [17]. The end-point trajectories were simulated with the following dynamical DOFs: shoulder flexion-extension, shoulder internal-external rotation, and elbow flexion-extension; other joints were assumed to be in the static neutral posture (the middle of the range of motion). The resulting N = 351 reaching movements were organized as the data structures containing [*q*,*q’*,*q’’*,τ], where *q* is the vector of joint angles, *q’* and *q’’* are its derivatives, and τ is the joint torque. Zero velocity and acceleration values of static DOFs were excluded from the state vector reducing 69 kinematic values to 27 values.

### Training, validation, and testing subsets

A total of 1,124,000 state vectors, representing all possible movement combinations, were split into training (71%), validation (15%), and testing (14%) subsets. We used one input sequence length (100 samples, fetched from state vectors) to train ANNs and additional three lengths (10, 20, and 50 samples) to examine the relative performance of transformations. The accuracy of the ANNs during the training process was monitored with a validation subset. The testing subset was unseen by ANN during the training and was used post hoc for the same purposes. Data representing different movement durations were equally distributed among the subsets. The z-score normalization was applied to input torque values to ensure their scaled contribution to ANN predictions. The predicted torques were then de-normalized to evaluate absolute errors across modeled DOFs.

### Hyperparameters

A preliminary search with a grid pattern was performed over four hyperparameters: learning rate (0.0001, 0.0005, or 0.001), the hidden layer size (23, 69, or 115), the number of recurrent layers (1, 3, or 5), and the network architecture type. The search resulted in 54 experiments that were limited to a training duration of only 15 epochs (each epoch is the use of a full training dataset). We considered the following three ANN types: Elman RNN [18], long short-term memory cell (LSTM) [19], and gated recurrent unit (GRU) [20], excelling at statistical forecasting [21].

### Statistical analysis

The mean squared error (MSE) between the target and predicted torques was used to monitor ANN training. The final accuracy was measured with root MSE (RMSE). The normality was tested with a one-sample Kolmogorov-Smirnov test, and the Kruskal-Wallis test was used to evaluate differences in non-normally distributed groups. The ANN execution time was measured on CPU and GPU using the Google Colab Pro environment. The ANN resistance to numerical noise was tested by perturbing test data with Gaussian noise. All analyses were conducted using PyTorch [22] and SciPy 1.0 [23] software with the significance level (α) set to 5%.

## Results

### Hyperparameter tuning

Effective training and accurate approximations require the optimization of many hyperparameters. To focus on the appropriate domain, we performed a preliminary hyperparameter grid search (see *Hyperparameters* in Methods). We found that the most critical hyperparameter was the learning rate. Consistent with the previous work [24], the use of slower values below 5×10^−4^ was less optimal–decreasing accuracy gain per epoch and increasing the training time. The learning rates over 5×10^−4^ resulted in less accurate and less dynamically stable solutions. The next parameter addressed in preliminary optimization was the number of hidden layers. There was no benefit to the deep structure of ANN, which resulted in the exclusion of most three- and five-layer configurations that suffered from overfitting and high inaccuracies in testing conditions.

### Approximating inverse dynamics

**Fig 2:**
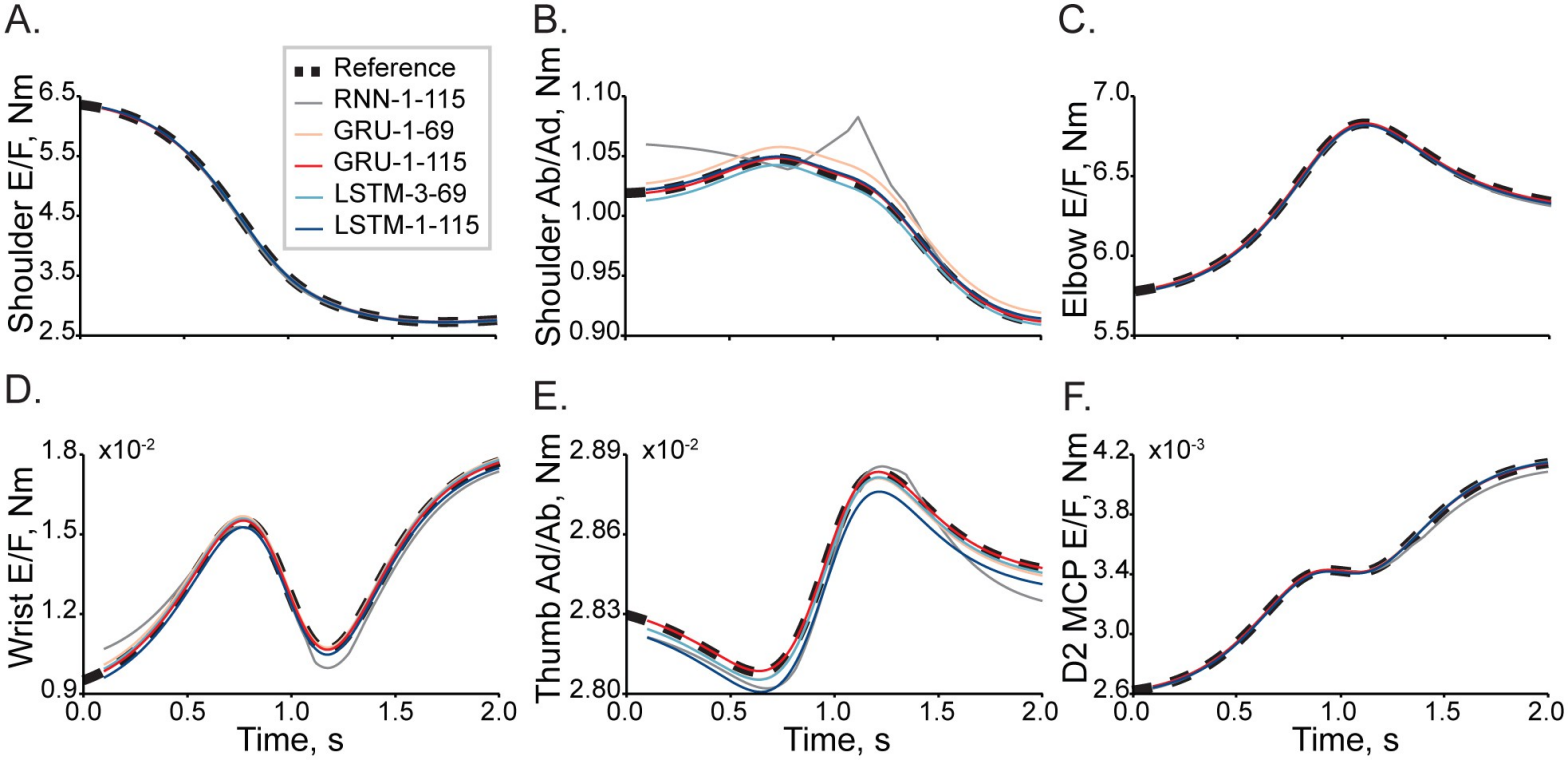
Selected example movement with the lowest errors of inverse dynamic transformation in a single trial. The performance of multiple ANNs is plotted as representative joint torques: (A) shoulder flexion, (B) shoulder adduction, (C) elbow flexion, (D) wrist flexion, (E) thumb abduction, and (F) index finger flexion. The used ANN naming convention is XXX-Y-ZZZ, where XXX is network type, Y is the number of hidden layers, and ZZZ is the number of the computational nodes within one hidden layer. The dashed reference trajectories represent torques calculated using the standard biomechanical model and indicate desired solutions to the inverse dynamics problem.

**Fig 3:**
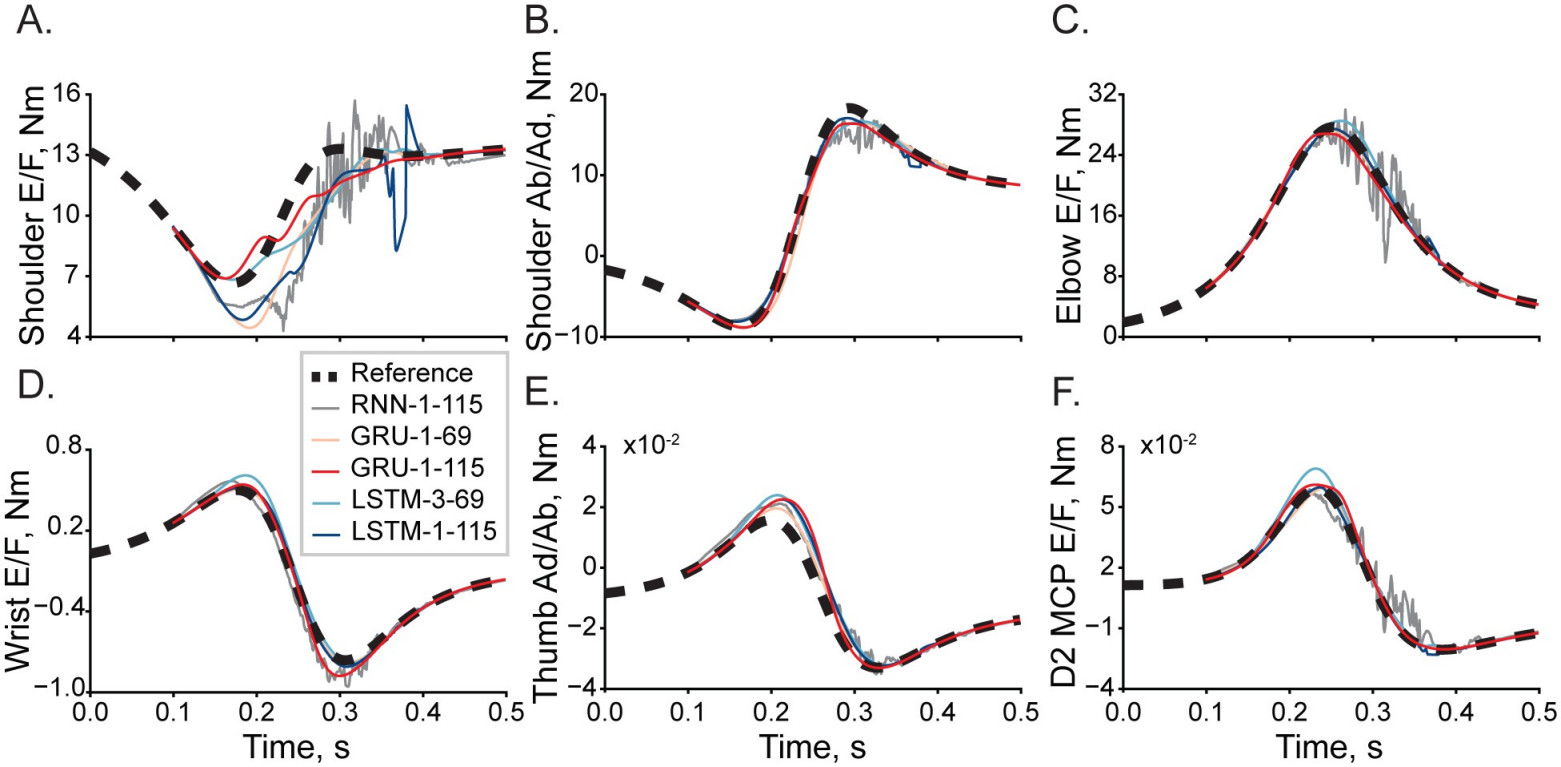
Selected example with the highest errors of inverse dynamic transformation in a single trial. The descriptions are the same as in Fig 2.

**Fig 4:**
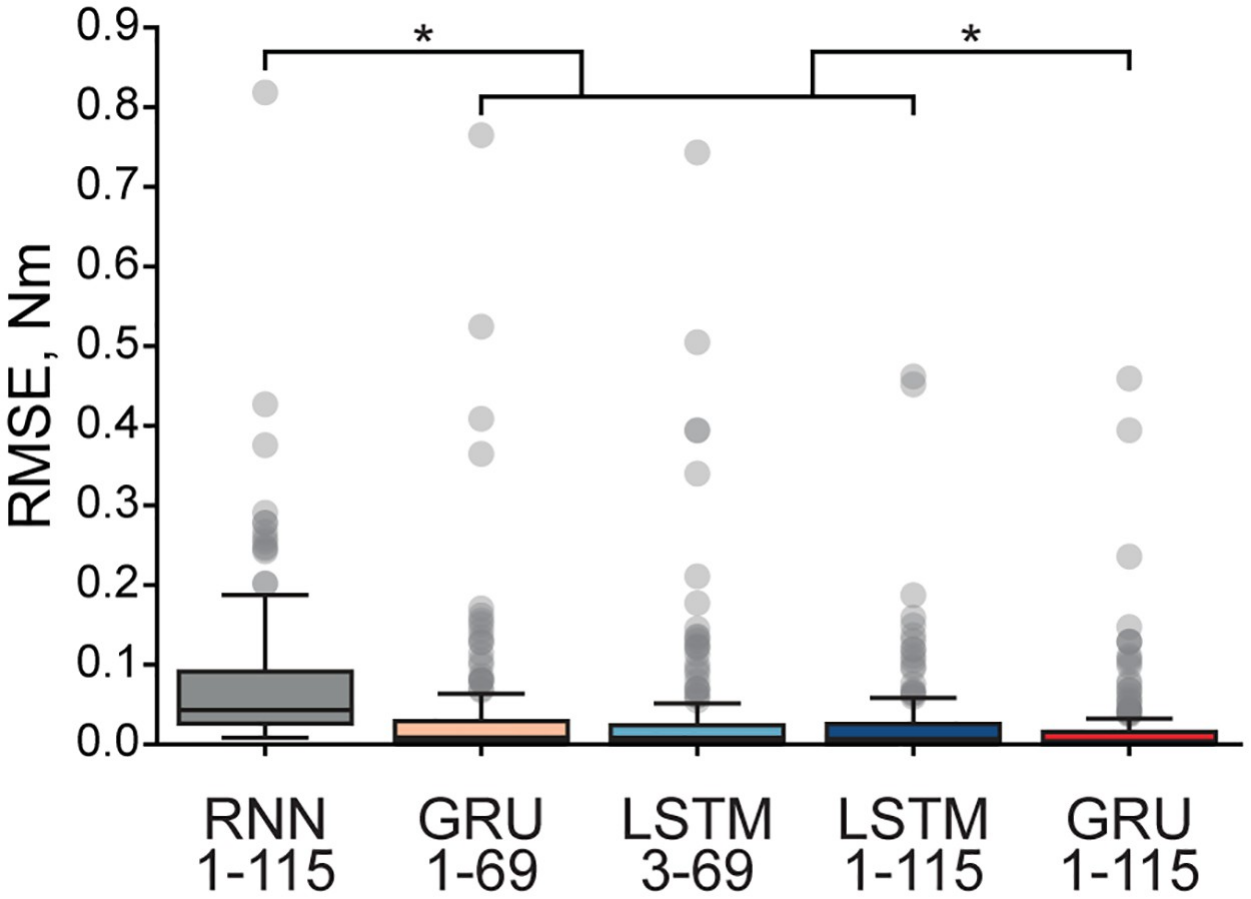
Simulated torque errors obtained for different ANN architectures. The box plots show the 25th and 75th percentiles of the RMSE distributions between target and simulated torques, solid horizontal lines are medians; whiskers indicate the full range, and circles mark outliers (1%). The significant differences tested with the Mann-Whitney pairwise comparison (*α* < 5%) are indicated with *.

Five candidate ANNs approximated solutions to the equations of motion describing the reaching movements in the 23 DOF arm and hand model. For all networks, the optimal learning rate was equal to 5×10^−4^. Fig 2 shows the best examples of a single-trial simulation with the lowest errors. Torques of proximal and distal joints show a close relationship with the reference trajectories (dashed). Contrasting these results to the worst performance shown in Fig 3, not all ANNs performed equally. The torque trajectories computed with the Elman RNN were prone to oscillatory behavior, as seen in Fig 3A, 3C and 3F. Similarly, the single-layer LSTM could generate large transient deviations, as seen in Fig 3A. Both Elman RNN and LSTM transformations were prone to amplitude scaling errors. The synthesis of performance across all trials in the test dataset is reported in Table 1. The best accuracy was attributed to the one-layer GRU with 115 computational nodes compared to other tested architectures (p<5×10^−6^, Fig 4). Elman RNN expectedly performed least accurately as compared to other ANNs for 100 sample sequences (p<7×10^−21^).

**Table 1:** The relationship of input length with accuracy for different ANN types. The most accurate candidates are marked in **bold**.

ANN Architecture	Torque RMSE, Nm			
	10 samples	20 samples	50 samples	100 samples
RNN-1-115	0.2870	0.1180	0.0757	0.0736
GRU-1-69	0.4515	0.3094	0.1149	0.0433
LSTM-3-69	1.1822	0.4339	0.0780	0.0398
GRU-1-115	0.3503	0.1837	0.0633	**0.0273**
LSTM-1-115	**0.2764**	**0.0872**	**0.0401**	0.0312

### Input sequence length

We used one input sequence length (100 samples) to train ANNs and tested them with four lengths (10, 20, 50, and 100) to examine the relative performance of transformations. The accuracy increased with the increase of sequence length, as shown by the mean predicted torque RMSE values in Table 1. This trend saturated for sequences of 50 samples and longer, resulting in <0.1 Nm errors for all ANNs. At shorter lengths (*n* = 20), RNN transformation outperformed GRU and the three-layer LSTM transformations. However, its performance did not improve as quickly as with other ANN types with longer sequences. Overall, both one-layer LSTM and GRU with 115 nodes delivered the highest accuracy.

### Computation latency

We measured the transformation latency per one output sample required by each ANN type to evaluate the real-time readiness of the current implementation using CPU and GPU hardware. On the GPU, one-layer ANNs processing up to 50 sample sequences were executed faster than real-time (<1 ms per sample, see S1 Fig). For implementations with 100 sample sequences, the latency declined to just under 2 ms per sample (see S1 Table). The three-layer LSTM was faster than real-time only in the 10-sample mode and took about 4 ms per sample in the 100-sample mode. On the CPU, one-layer ANNs were executed in real-time only with 10 sample sequences, except for Elman RNN, which maintained real-time performance with 20 sample sequences. For the mode with 100 samples, the latencies were about 5.5 ms per sample, and the Elman RNN was the fastest executing at 2 ms per sample. The three-layer architecture was slow in 10 (~2 ms) and 100 (~14 ms) sample modes.

### Numerical noise

We examined ANN tolerance to noise in models with 100 sample input sequences by adding 1% and 5% of peak-to-peak Gaussian noise to kinematic trajectories (Table 2). The accuracy of Elman RNN decreased 1.4x with 1% noise and 4.06x with 5% noise relative to simulations without noise. The accuracy decreases were 1.42x and 4.22x for the 69-node GRU and 1.6x and 5.25x for the 115-node GRU. The absolute error values, however, were lower in GRU 1–115 than those in GRU 1–69 with noise. The LSTM transformations were the most sensitive to noise, with a decrease of 2.48x and 8.86x for the single-layer structure and a decrease of 11.98x and 30.28x for the triple-layer structure.

**Table 2:** The relationship between input noise and accuracy for different ANN types. The solutions with the highest noise tolerance are indicated in **bold**.

ANN Architecture	Torque RMSE, Nm	
	1% Noise	5% Noise
RNN-1-115	0.103	0.2987
GRU-1-69	0.0616	0.1827
LSTM-3-69	0.4770	1.2050
GRU-1-115	**0.0438**	**0.1433**
LSTM-1-115	0.0775	0.2765

## Discussion

In this study, we developed and tested multiple ANN architectures to solve the spatiotemporal inverse dynamics of a segmented arm and hand. The numerical model of arm dynamics was used to create input-output datasets for the physiological workspace of arm movements. The trained ANNs were examined for accuracy, robustness, and execution efficiency to validate the ANN approach and to examine the impact of different parameters. We demonstrate that the single-layer LSTM and GRU models with 115 nodes can perform accurate and real-time simulations. These simulations executed on a cloud service GPU with 50 ms sequences produce the most accurate results. The same models can run in real-time on a cloud service CPU with short sequences (10 samples) and, consequently, less accuracy. The additional testing with noisy inputs demonstrated the robustness of models to resist noise generating less than 0.05 Nm errors with the additional 1% noise that increased three-fold with the additional 5% noise (see Table 2, GRU model).

### Optimal network structure

We have conducted a preliminary parametric search of ANN structures simulating high dimensional (23 DOF) inverse dynamics of the human arm and hand. This limited our analysis to five candidates that were expected to generate *“good-enough”* approximations. Figs 2 and 3 show the performance of the ANNs. While the performance across all ANNs was surprisingly accurate, the Elman RNN model generated oscillatory predictions suggesting that the training might have reached a suboptimal solution near a local minimum, for example, near saddle-type critical points. With the increasing realism in the biomechanics of human limbs, these saddle points proliferate, presenting a challenge to ANN training [25]. The methods to overcome it exist [26]; however, they have not been implemented in modern programming frameworks. A second issue with the Elman RNN model was the saturation of performance when the training MSE approached zero. This problem, termed the “vanishing gradients”, is apparent in the relatively high errors in Fig 4. GRU and LSTM model types were not prone to this methodological limitation. In LSTM models, scaling errors were minimal; however, the outcomes had infrequent dynamical transients (e.g., Fig 3A), which would be destabilizing in forward dynamic simulations. These transients indicate the overfitting that occurs in LSTMs with many computational nodes [27]. The LSTM with fewer nodes did not suffer from this problem in support of this idea. The GRU model outputs were smooth with only minor scaling and dynamical errors. This high performance is likely a result of the algorithmic complexity of the GRU, which falls between that of the Elman RNN and the LSTM and mitigates diminishing returns of both simpler and more complex architectures in this problem, effectively minimizing the probability of overfitting [28]. Overall, the one hidden layer GRU was the most accurate with LSTM as a close second indicating that GRU and LSTM topologies are best suited for solving inverse dynamics for realistic limb biomechanics.

### Sequences for neural net training

Many behaviors are dynamical, history-dependent, and produced by sequences of actions. All goal-directed movements are a result of sequential and coordinated actions of the musculoskeletal system. For example, the actions of walking and reaching to a target are generated by the spatiotemporal sequences of cortical ensembles and muscle groups generating forces that result in the progression of mechanical actions [29]. The planning and execution within neural control pathways are thought to require both inverse and forward computations to generate appropriate control sequences and to monitor their execution through the comparison of expected and sensed signals. This general embedding of control structure within neural pathways is supported by the theory of internal models [30, 31]. Historically, the ANN approach was envisioned as the simplified formulation of neural computations [32], but the current implementations are limited compared to neural functions [33]. For ANNs to solve the control of movement, the essential computations need to solve musculoskeletal limb kinematics and history-dependent limb dynamics.

Our result demonstrates that the use of sequences in training and evaluation of ANN models for inverse dynamics is a valid approach. LSTM and GRU models for natural language processing [34, 35] and kinematic video tracking [36] embed history-dependent relationships within sequences and are less sensitive to the problem of the “catastrophic forgetting” [37]. Similarly, the Newtonian relationship through second-order differential equations governing joint movement imposes the history dependence on kinematic sequences. This study provides the first demonstration of realistic arm and hand dynamics with the use of ANNs with memory capabilities to represent sequences.

We extend the previous studies focused on relatively low-dimensional dynamics of industrial robotic systems to realistic high-dimensional arm and hand movements. Previously, inverse dynamic transformations with ANNs were typically limited to several degrees of freedom for the control of robotic devices (e.g., Kuka arm robot, 5–7 DOFs) [38, 39]. Yet, we achieve similar torque prediction performance (less than 0.15 Nm errors with a high level of noise) as in these low-dimensional systems [40]. Furthermore, we propose this approach as the extension for the full musculoskeletal dynamics that details moment arm and muscle length relationships with postures [3]. This combination of physically explainable ANN models can, thus, provide decoding of motor intent or the control of wearable powered robotics using a muscle-level resolution of biomechanical state.

## Limitations

We chose to perform a heuristic grid-search method to identify essential hyperparameters and select them for further testing. Yet, automating the design of ANN can produce solutions outperforming custom architectures [41, 42]. For example, the optimization of hidden layers and nodes [43] within the optimization of accuracy and decreased model complexity can find ANN structures that outperform the current result. Overall, the full parameter space is currently unexplored for the applications that describe limb dynamics. Another potential limitation is the limited exploration of behavioral space in this and other previous studies. While we have introduced a generalized approach to the representation of the workspace for reaching movements, other behavioral tasks may be challenging for this model. For example, grooming, object interactions, or pathologies like tremor (high-speed 8 Hz oscillations) may need to be included in the training dataset for specific applications. The movements with sharp direction reversals may require models with shorter than 50 ms (Table 2) input sequences. Yet, we showed that a single-layer LSTM could maintain prediction accuracy (about 0.1 Nm error) with only 20 ms input sequences indicating that the proposed method remains valid with the outlined mitigation strategies.

## Conclusions

In this study, we trained and optimized a machine learning algorithm to approximate solutions to the complex inverse dynamic problem. The ANNs were tested on a 23-DOF human arm and hand model. We demonstrated that simple one-layer ANNs with memory are real-time accurate and noise-tolerant when predicting kinetics in complex multi-joint systems. Together, these findings suggest an instrumental role for ANNs in the development of control algorithms that approximate dynamic simulations with minimal latencies, thus enabling accurate and computationally inexpensive control of wearable prosthetics and assistive devices.

## Supporting information

S1 Fig Average forward propagation latencies as a function of sequence length. (A) On the GPU, one-layer ANNs processing up to 50 sample sequences were faster than real-time, taking less than 1 ms per sample. However, when the number of sample sequences increased to 100, the latency increased to just under 2 ms per sample. The three-layer LSTM network achieved faster-than-real-time performance only with 10-sample sequences, while it took approximately 4 ms per sample in the 100-sample mode. On the CPU, real-time execution of one-layer ANNs was possible only with 10 sample sequences, except for the Elman RNN, which maintained real-time performance with 20 sample sequences. In the case of the 100-sample mode, the latencies increased to approximately 5.5 ms per sample, with the Elman RNN being the fastest, executing at 2 ms per sample. The three-layer architecture exhibited slower performance, requiring approximately 2 ms per sample in the 10-sample mode and around 14 ms per sample in the 100-sample mode. The used naming convention is XXX-Y-ZZZ, where XXX is ANN type, Y is the number of hidden layers, and ZZZ is the number of the computational nodes within one hidden layer; type “RNN” refers to Elman RNN.
(PDF)

S1 Table Average latencies for the forward propagation of the input sequence consisting of 100 samples. (DOCX)
