# Supplementary figures and images for: Artificial physics engine for real-time inverse dynamics of arm and hand movement
Source: PLoS One. 2023 Dec 13;18(12):e0295750. doi: 10.1371/journal.pone.0295750 (PMC10718432; doi:10.1371/journal.pone.0295750)

A.

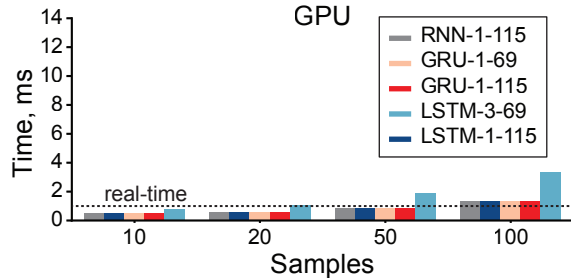

B.

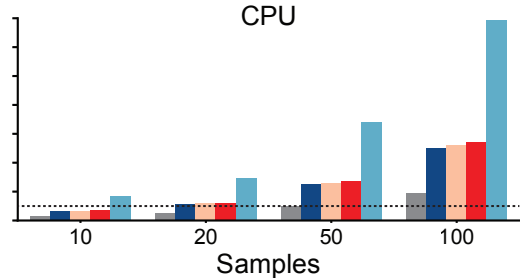

Supplement: S1 Fig — (A) On the GPU, one-layer ANNs processing up to 50 sample sequences were faster than real-time, taking less than 1 ms per sample. However, when the number of sample sequences increased to 100, the latency increased to just under 2 ms per sample. The three-layer LSTM network achieved faster-than-real-time performance only with 10-sample sequences, while it took approximately 4 ms per sample in the 100-sample mode. On the CPU, real-time execution of one-layer ANNs was possible only with 10 sample sequences, except for the Elman RNN, which maintained real-time performance with 20 sample sequences. In the case of the 100-sample mode, the latencies increased to approximately 5.5 ms per sample, with the Elman RNN being the fastest, executing at 2 ms per sample. The three-layer architecture exhibited slower performance, requiring approximately 2 ms per sample in the 10-sample mode and around 14 ms per sample in the 100-sample mode. The used naming convention is XXX-Y-ZZZ, where XXX is ANN type, Y is the number of hidden layers, and ZZZ is the number of the computational nodes within one hidden layer; type “RNN” refers to Elman RNN. (PDF) [file pone.0295750.s001.pdf]
