# Supplementary material for: Artificial physics engine for real-time inverse dynamics of arm and hand movement
Source: PLoS One. 2023 Dec 13;18(12):e0295750. doi: 10.1371/journal.pone.0295750 (PMC10718432; doi:10.1371/journal.pone.0295750)
Supplement: S1 Table — (DOCX) [file pone.0295750.s002.docx]

**S1 Table Average latencies for the forward propagation of the input sequence consisting of 100 samples.**

|  | Latency, ms | | | | |
| --- | --- | --- | --- | --- | --- |
| Device | RNN-1-115* | GRU-1-69 | LSTM-3-69 | GRU-1-115 | LSTM-1-115 |
| CPU | 1.971 | 5.219 | 13.886 | 5.459 | 5.025 |
| GPU | 1.354 | 1.356 | 3.337 | 1.354 | 1.379 |

*Used naming convention is XXX-Y-ZZZ, where XXX is ANN type, Y is the number of hidden layers, and ZZZ is the number of the computational nodes within one hidden layer; type “RNN” refers to Elman RNN.
